# A Case of a Heterozygous Female Patient With Ornithine Transcarbamylase (OTC) Deficiency Successfully Treated by Liver Transplantation in Adulthood

**DOI:** 10.7759/cureus.87892

**Published:** 2025-07-14

**Authors:** Rieko Kosugi, Takeshi Usui, Tatsuhide Inoue, Hiroyuki Ariyasu

**Affiliations:** 1 Endocrinology, Diabetes and Metabolism, Shizuoka General Hospital, Shizuoka, JPN; 2 Medical Genetics, Shizuoka Graduate University of Public Health, Shizuoka, JPN

**Keywords:** heterozygous female, living donor liver transplantation, metabolic diseases, ornithine transcarbamlyase deficiency, pediatric to adult transition

## Abstract

Ornithine transcarbamylase deficiency (OTCD) is a urea cycle disorder inherited in an X-linked manner. This report describes a case of a 27-year-old woman diagnosed with OTCD during childhood who experienced growth disturbances and an unstable clinical condition (nausea, vomiting, and hyperammonemia) despite strict dietary and pharmacological management, leading to a poor quality of life. She met the scoring criteria for liver transplantation (LT), received a living donor liver transplant (LDLT) from her mother, and is doing well post-transplant. LT is the only curative treatment available for OTCD, and recent technological advances have significantly improved outcomes in Japan. However, as female patients with OTCD were usually considered to have a mild phenotype, the induction and timing of LT are very difficult to decide. In addition, most LT in female patients with OTCD is performed in childhood; therefore, there are very few case reports of female OTCD patients with LT in adulthood. This case study provides guidance on the indications for LT in adult female OTCD patients.

## Introduction

Urea cycle disorders (UCDs) represent a group of inherited metabolic disorders characterized by impaired nitrogen detoxification caused by defects in urea cycle enzymes. Ornithine transcarbamylase deficiency (OTCD, OMIM #300461) is the most common UCD, with an estimated prevalence of 1 in 80,000 births. The responsible gene is *OTC*, which is located on chromosome Xp11.4 [1,2].

OTCD often manifests with a life-threatening metabolic crisis of hyperammonemia. Clinical symptoms are usually nonspecific and neurological, including nausea, vomiting, convulsions, altered consciousness, and developmental issues. To avoid hyperammonemia, lifelong management is essential. It involves dietary protein restriction, medications to increase waste nitrogen excretion (such as sodium phenylbutyrate and sodium benzoate), and supplementation with arginine, citrulline, essential amino acids, and carnitine [3,4].

The severity of female subjects with OTCD varies due to inactivation of the X chromosome. Contrary to previous findings that heterozygous female patients show a mild phenotype, recent reports have shown that 22% of them are clinically affected, with a mortality rate of 4% [5]. Management of plasma ammonia (NH_3_) levels requires lifelong strict dietary restrictions and pharmacotherapy, severely impairs quality of life, and constantly induces fear of attacks. Liver transplantation (LT) should be considered in female patients with symptomatic OTCD. However, the criteria for LT in female patients with OTCD are not clear, and LT is mainly performed in childhood. Therefore, in most cases, especially in adulthood, pharmacological and dietary therapies remain the primary treatment options.

## Case presentation

The patient, a 27-year-old woman, was born following a 41-week gestation period through vaginal delivery, weighing 3010 g without any perinatal abnormalities. At the age of two years and 11 months, she experienced drowsiness due to hyperammonemia and was subsequently diagnosed with OTCD. Markedly elevated plasma glutamine (Gln) and excessive urinary excretion of orotic acid and uracil at age 2, as detailed in the left-hand column of Table 1, led to the diagnosis of OTCD.

**Table 1:** Laboratory and nutritional findings at three time points. Data are presented for age 2 (onset, left column), age 18 (initial visit, middle column), and age 30 (post-liver transplantation, right column). At the onset, the diagnosis was based on elevated ammonia and glutamine levels and excessive urinary orotic acid and uracil. NH₃ and Gln remained slightly high at the initial visit, and nutritional intake was insufficient. Post-transplantation, both parameters and nutritional status improved. The reference in the nutritional states section indicates the target energy and safe protein intake levels for adults with OTCD. NH₃, ammonia; Arg, arginine; Cit, citrulline; Gly, glycine; Gln, glutamine; Ile, isoleucine; NA, not available; OTCD, ornithine transcarbamylase deficiency

Parameter	Age 2 (At Onset)	Age 18 (First Visit)	Age 30 (After LT)	Unit	Reference Range
A. Laboratory findings					
Blood Biochemistry					
NH_3_	364	117	29	μg/dL	12-66
Gln	1944	1349	604	μmol/L	503.4-851.4
Arg	5.3	92	93.3	μmol/L	44.1-115.2
Ile	NA	51.2	102	μmol/L	44.9-120.3
Gly	NA	282.7	264.1	μmol/L	136.8-397.7
Cit	18.8	19.7	15	μmol/L	18.2-50.1
Urine Biochemistry					
Orotic acid	228.93	NA	NA	μmol/gCre	NA
Uracil	54.1	NA	NA	μmol/gCre	NA
B. Nutritional status					
Energy	NA	35	50	kcal/kg/day	43 (50 kg Body Weight)
Protein	NA	0.5	1.7	g/kg/day	0.83

**Figure 1:**
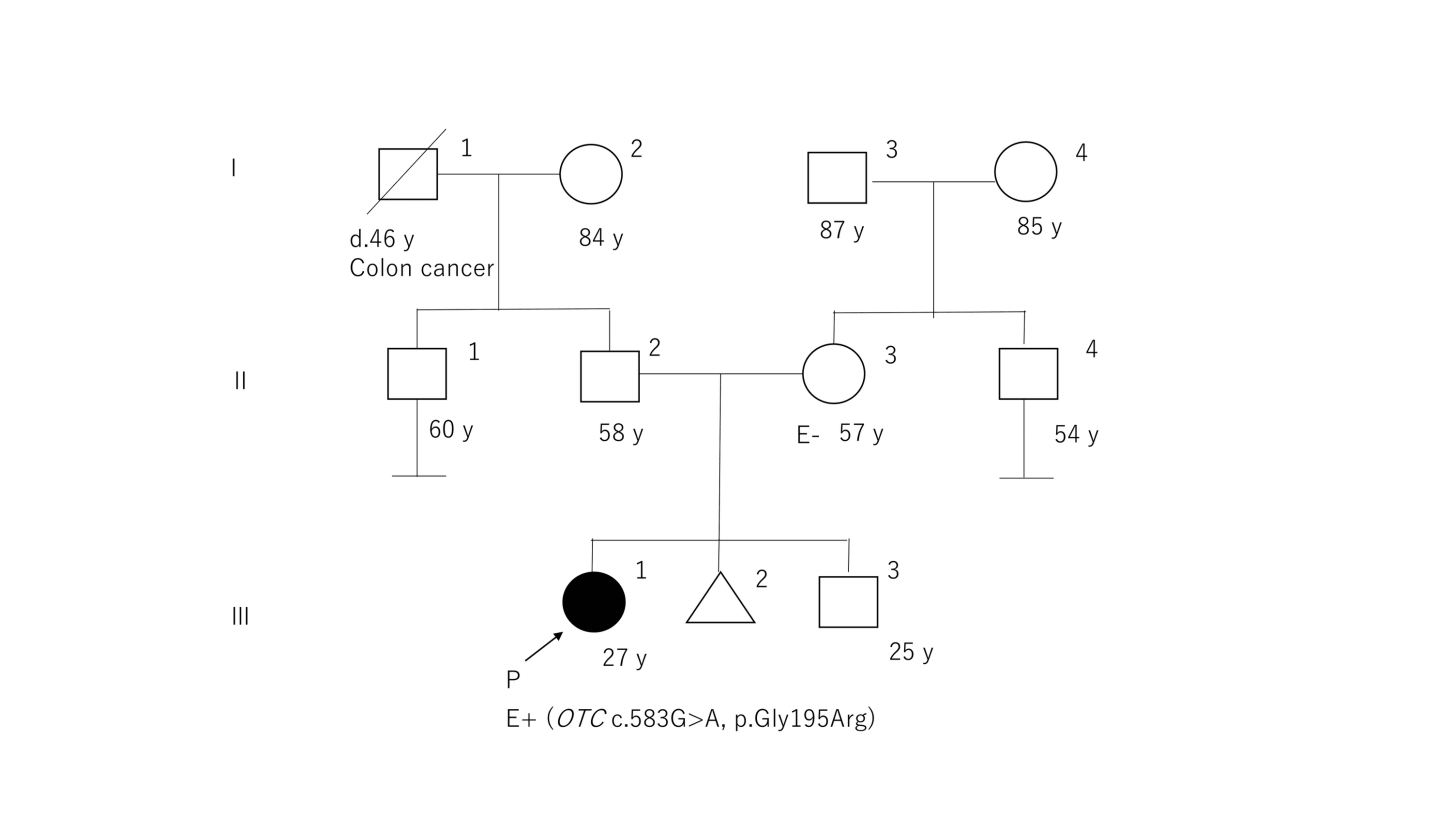
Family pedigree. Squares and circles represent male subjects and female subjects, respectively. Black symbols indicate individuals carrying the disease-causing variant (*OTC *c.583G>A, p.Gly195Arg). No individuals other than the proband had a clinical phenotype suggestive of OTCD. OTCD, ornithine transcarbamylase deficiency

In the chronic phase, she exhibited cognitive impairments, with a full-scale IQ of 83 (verbal IQ: 68, performance IQ: 56) assessed at 13 years. No family members exhibited symptoms suggestive of OTCD (Figure 1). 

**Figure 2:**
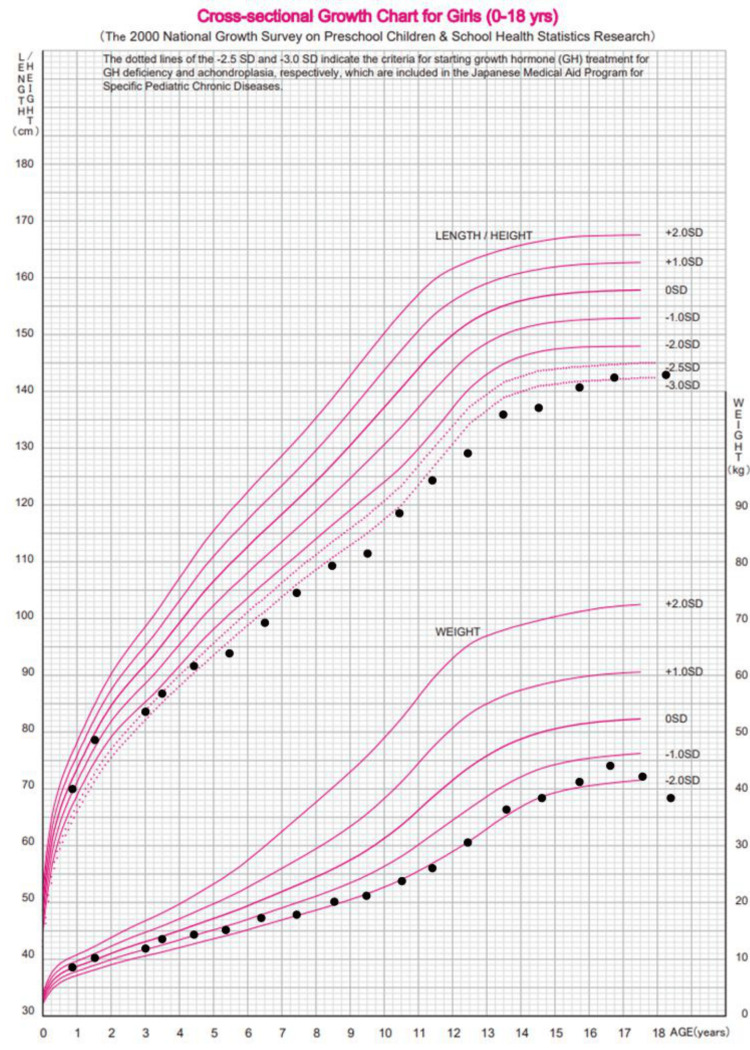
Patient’s growth trajectory, represented by black dots, overlaid on standard growth curves for Japanese girls from the 2000 National Survey. Growth trajectory of the patient overlaid on cross-sectional growth charts for Japanese girls aged 0–18 years. The patient’s height and weight at various ages (black dots) are plotted on standard reference curves based on the 2000 National Growth Survey in Japan [6]. Following disease onset, a gradual decline in growth parameters was observed, with values falling below −2 standard deviations.

From childhood, she received consistent dietary management and pharmacotherapy, which successfully prevented any emergency state. At 18, she sought care at our hospital for transition. Figure 2 illustrates the patient’s growth trajectory up to the initial visit. Laboratory findings and nutritional status at age 18 are summarized in the middle column of Table 1. 

**Figure 3:**
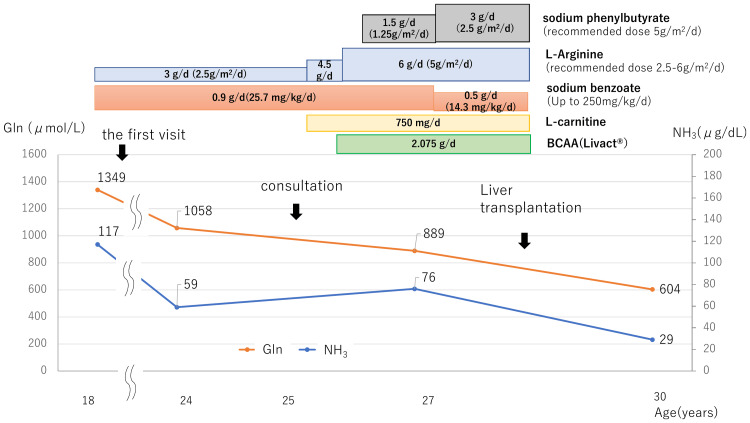
Clinical course before and after liver transplantation. The figure illustrates the longitudinal changes in plasma NH_3_ and Gln levels in response to medical management prior to liver transplantation. With intensified pharmacological therapy, both parameters gradually approached the target range. All medical treatments were discontinued after transplantation, and both values remained within the normal range. BCAA: branched-chain amino acid; Gln: Glutamine; NH_3_: ammonia

At the first visit, she was underweight (-2.3 SD) and short (-2.5 SD), with mildly elevated NH_3_ levels (117 µg/dL, 68.7 µmol/L: reference range 12-66 µg/dL, 6.7-38.8 µmol/L) but elevated plasma Gln levels (1349 µ mol/L: reference range 50.3-851.4 µ mol/L). Her protein (0.5 g/kg/day: safe level of intake 0.83 g/kg/day) and energy intake (35 kcal/kg/day: energy requirement 43 kcal/kg 50kgBW) were below the recommended levels for her age. The management targets in OTCD are NH_3_ levels below 140 µg/dL (80 µmol/L) and plasma Gln levels below 1000 µmol/L. To achieve the target range, she was put on a strict diet for seven years from the time of her first visit, and her weight gradually declined from 38 kg to 34 kg. At the age of 25, we consulted a specialist in inherited metabolic diseases for optimal management to enhance her nutritional status and quality of life. Changes in laboratory data and medication regimens are shown in Figure 3. Sodium benzoate, a nitrogen scavenger facilitating NH_3_ excretion via an alternative route, is not a registered drug. Therefore, the approved sodium phenylbutyrate was substituted, and the dose was increased to the maximum tolerated dose without side effects (2.5 g/m^2^/day, recommended dose 5 g/m^2^/day). In addition, the dose of L-arginine was maximized (5 g/m²/day, 6 g/day: recommended dose 2.5-6 g/m²/day, max 6 g/day), and L-carnitine was administered at 750 mg/day. The dose of L-carnitine was adjusted by monitoring serum and urine free carnitine levels. Branched-chain amino acids (BCAAs) (LIVACT Combination Granules® 2.075 g/day) were supplemented to supplement protein intake from the diet. As a result, usual plasma NH_3_ levels were within the target range (76 μg/dL), and Gln levels decreased (889 µmol/L). However, she presented with transient hyperammonemia with gastrointestinal symptoms depending on her diet, resulting in her continuing to restrict her diet and sustained weight loss.

Her quality of life was severely impaired despite receiving the maximum tolerated doses of pharmacological therapy due to strict dietary protein restrictions aimed at controlling severe hyperammonemia. Furthermore, it is well recognized that baseline NH_3_ levels can increase before the onset of severe seizures in OTCD, and this patient was considered to be at high risk of developing such life-threatening episodes.

The patient and her mother strongly wished to be freed from the burdens of protein restriction and the constant fear of seizures, and thus, they expressed a strong desire for LT to improve their quality of life. She was eligible for LT based on Japan’s Ministry of Health, Labour, and Welfare scoring system for metabolic diseases. After discussions with transplant surgeons and specialists in congenital metabolic disorders and following informed consent, she underwent an LDLT from her mother.

Prior to the surgery, genetic testing was performed on both the patient and her mother. The patient possessed a pathogenic variant in the *OTC* gene (NM_000531.6 c.583G>A, p.Gly195Arg), whereas her mother did not have this variant. It is presumed that the disease-causing variant had arisen de novo [7]. At the age of 27, she underwent an LT using a left hepatic lobe graft. Postoperatively, she remained hospitalized for three months, experiencing ascites due to mild acute cellular rejection. She was treated with prednisolone and tacrolimus for one year. Currently, her condition is stable with tacrolimus therapy (5 mg/day) only; she no longer faces dietary protein restrictions. The right column of Table 1 shows her post-LT amino acid profile and dietary intake. In particular, her plasma NH_3_ and amino acid levels have largely normalized, despite significantly increasing her calorie and protein intake. As a result, her quality of life has improved significantly, and she is still progressing well without complications four years after LT.

The patient was informed that data concerning the case would be submitted for publication and provided consent.

## Discussion

OTCD, an X-linked recessive genetic disorder, leads to severe symptoms in hemizygous male subjects from the neonatal period, often resulting in a fatal outcome without treatment. In contrast, female subjects exhibit a range of symptoms from asymptomatic to severe, depending on the degree of X-chromosome inactivation [8,9]. It was previously believed that most female patients with OTCD cases could be managed with nutritional and pharmacological therapies. However, a recent cohort study of 289 female patients with OTCD revealed that 64 (22%) were clinically affected. These included neonatal type (10 female patients, 3.5%), acute episodic type, such as consciousness disturbances after the neonatal period (32 female patients, 11%), and chronic type (22 female patients, 7.5%). The overall mortality rate among symptomatic female subjects was 19% (12/64), with specific rates of 30% (3/10) for neonates, 22% (7/32) for episodic type, and 9% (2/22) for chronic type [5]. Consequently, severe conditions and even death in female patients with OTCD are not uncommon. The primary treatment modalities include nutrition and pharmacological therapy, yet LT should also be considered for female patients, as demonstrated in this case.

In Japan, indications for LT in OTCD differ between the acute and chronic phases [10]. In the acute phase, the indication is for patients who cannot be weaned off hemodialysis and whose lives cannot be saved except by LT. In the chronic phase, on the other hand, cases with frequent acute exacerbations despite standard management, poor adherence, growth failure, or strict dietary restrictions are mainly eligible for LT to improve long-term prognosis and quality of life. International guidelines for UCDs also consider severe UCDs that do not respond adequately to standard treatment or have poor quality of life as candidates for LT, which is similar to the criteria in Japan [3]. The indication for LT is graded by a scoring system based on guidelines issued by Japan’s Ministry of Health, Labour and Welfare (MHLW). This system evaluates disease specificity, effectiveness of conventional medical care, quality of life, growth and development, and laboratory findings [11,12]. A cumulative score of 10 or above is considered a definitive indicator of transplant eligibility (Table 2). In this case, the patient and her family strongly desired LT to improve her quality of life, and she was considered eligible based on a total score of 11.

**Table 2:** LT scoring based on Japan’s Ministry of Health, Labour and Welfare (MHLW) guidelines for inherited metabolic diseases. The criteria include disease specificity, efficacy of medical therapy, quality of life, current clinical condition, and laboratory findings. Black filled circles indicate the categories applicable to this patient. The total score was 11, meeting the recommended threshold for LT indication. LT: Liver transplantation

Category	Item	Score 5	Score 3	Score 1
Disease specificity	Is the metabolic abnormality limited to the liver?	●		
	Are there previous reports of liver transplantation for this condition?		●	
Efficacy of medical therapy	Metabolic decompensation requiring hospitalization (≥6 times/year)	○		
	Metabolic decompensation requiring hospitalization (3–5 times/year)		○	
	Metabolic decompensation requiring outpatient care (≥6 times/year)			●
	Metabolic decompensation requiring apheresis or ICU care (excluding initial onset; ≥2 times/year)	○		
	Severely poor compliance with dietary and pharmacological therapy		○	
	Poor compliance with dietary and pharmacological therapy			○
Quality of life (QOL)	Tube feeding or frequent meals (if improvement expected)		○	
	Prevention or improvement of neurological impairment		○	
Current condition	Neurological status (developmental): Able to perform some daily activities			●
	Physical growth status: Growth impairment (height < -2.5 SD)			●
Laboratory findings	Persistent abnormal laboratory findings		○	

Japan differs significantly from other countries in that the majority of LT are LDLT due to the limited availability of deceased donors. Internationally, in regions such as Europe and North America, deceased donor liver transplantation (DDLT) is the mainstream procedure. In Japan, by 2022, 93 cases (including five adult patients) had undergone LDLT, with a cumulative survival rate of 96.8%, which was very favorable [13]. A multi-center cohort study from the UK reported a 95% overall survival rate in OTCD patients who underwent LT [14]. A European Society for the Study of Inborn Errors of Metabolism (SSIEM) report, focusing on female patients with OTCD, showed a 100% survival rate, with a median follow-up of 6.1 years [15]. These findings are generally consistent with data reported from Japan, demonstrating favorable outcomes for DDLT and LDLT. However, given the lifelong requirement for immunosuppressive therapy following LT, the technical complexity of the procedure, and the uneven distribution of transplant centers, the indication for LT cannot be determined solely based on existing scoring systems. It should also incorporate evidence from case reports, cohort studies, and expert consensus. Additionally, there are ethical issues related to donors in LDLT. Therefore, determining the indication and timing of LT, especially in female OTCD patients with heterogeneous phenotypes, is highly challenging, and multidisciplinary team discussions are essential [14]. In this case, the indication for LT was determined through discussions between experts in congenital metabolic disorders and transplant surgeons, and LT was performed after the risks were fully explained. Furthermore, to reduce perioperative mortality, it is necessary for patients to maintain a relatively stable metabolic state before LT [3,15]. However, most physicians have no experience treating OTCD and no reviews or detailed case reports showing specific medication adjustments for OTCD in adulthood. In the present case, preoperative Gln and NH_3_ levels were controlled with maximum pharmacological treatment, under the guidance of experts in congenital metabolic diseases.

Although the literature on long-term follow-up of LT in OTCD is limited, studies have shown that LT is beneficial in improving quality of life [11]. In a nationwide Japanese survey, 16 female patients with OTCD underwent LT. Long-term survival rates were higher in patients who underwent LT, demonstrating its effectiveness in relieving gastrointestinal symptoms such as nausea, vomiting, and neurological symptoms. Postoperatively, patients were relieved of protein restriction, and amino acid analysis showed near-normal levels, except slightly lower citrulline levels [16]. On the other hand, 82% of the patients in this cohort were children under 10 years of age at the time of LT transplantation, with no mention of adult female patients with OTCD. In this patient, plasma amino acid levels have normalized without dietary restriction or pharmacological therapy. The patient's food intake and weight increased, and her quality of life improved significantly.

Additionally, LT is beneficial for improving growth failure [17]. Previously, Nakamura et al. reported that the final height of female subjects with OTCD was 150.3 ± 7.2 cm (-1.48 SD) [18]. In contrast, the present case had a final height of 143 cm (-2.5 SD), likely due to difficulties adjusting medication during the growth period and inadequate nutritional intake resulting from strict dietary restrictions aimed at preventing hyperammonemia. Since the patient is already an adult, further height gain is not expected; however, improved nutritional status following LT may contribute to the prevention of sarcopenia in the long term. These results suggest that LT is a highly effective option in female patients with OTCD, even in adulthood, with the potential to improve quality of life, prevent acute episodes, and increase long-term survival.

## Conclusions

In conclusion, we present a rare case of a female patient with childhood-onset OTCD who underwent successful LT in adulthood after long-term pharmacological and dietary management. While the life expectancy of OTCD patients has improved with conventional therapies, the burden of chronic treatment often compromises the quality of life, particularly in adulthood. In cases where metabolic control remains suboptimal or quality of life is markedly impaired despite maximal medical therapy, LT should be actively considered as a curative option through multidisciplinary team discussion. Although reports of adult female patients with OTCD undergoing LT are limited, accumulating such cases is essential for refining treatment criteria and improving clinical decision-making. This case provides practical insight into the preoperative management and clinical indications for LT in adult female patients with OTCD.

## References

[1] Lindgren V, de Martinville B, Horwich AL, Rosenberg LE, Francke U (1984). Human ornithine transcarbamylase locus mapped to band Xp21.1 near the Duchenne muscular dystrophy locus. Science.

[2] Nagata N, Matsuda I, Oyanagi K (1991). Estimated frequency of urea cycle enzymopathies in Japan. Am J Med Genet.

[3] Häberle J, Burlina A, Chakrapani A (2019). Suggested guidelines for the diagnosis and management of urea cycle disorders: first revision. J Inherit Metab Dis.

[4] Matsumoto S, Häberle J, Kido J, Mitsubuchi H, Endo F, Nakamura K (2019). Urea cycle disorders-update. J Hum Genet.

[5] Gobin-Limballe S, Ottolenghi C, Reyal F (2021). OTC deficiency in females: phenotype-genotype correlation based on a 130-family cohort. J Inherit Metab Dis.

[6] Isojima T, Kato N, Ito Y, Kanzaki S, Murata M (2016). Growth standard charts for Japanese children with mean and standard deviation (SD) values based on the year 2000 national survey. Clin Pediatr Endocrinol.

[7] Tuchman M, Plante RJ, McCann MT, Qureshi AA (1994). Seven new mutations in the human ornithine transcarbamylase gene. Hum Mutat.

[8] Ricciuti FC, Gelehrter TD, Rosenberg LE (1976). X-chromosome inactivation in human liver: confirmation of X-linkage of ornithine transcarbamylase. Am J Hum Genet.

[9] Yorifuji T, Muroi J, Uematsu A (1998). X-inactivation pattern in the liver of a manifesting female with ornithine transcarbamylase (OTC) deficiency. Clin Genet.

[10] Japanese Society for Inherited Metabolic Diseases (2019). Newborn Mass Screening Disease Practice Guideline. https://jsimd.net/pdf/newborn-mass-screening-disease-practice-guideline2019.pdf.

[11] Wakiya T, Sanada Y, Mizuta K (2011). Living donor liver transplantation for ornithine transcarbamylase deficiency. Pediatr Transplant.

[12] Kasahara M, Sakamoto S, Horikawa R (2014). Living donor liver transplantation for pediatric patients with metabolic disorders: the Japanese multicenter registry. Pediatr Transplant.

[13] (2023). Liver Transplantation in Japan Registry by the Japanese Liver Transplantation Society (Article in Japanese). Jpn J Transplant.

[14] Seker Yilmaz B, Baruteau J, Chakrapani A (2023). Liver transplantation in ornithine transcarbamylase deficiency: a retrospective multicentre cohort study. Mol Genet Metab Rep.

[15] Molema F, Martinelli D, Hörster F (2021). Liver and/or kidney transplantation in amino and organic acid-related inborn errors of metabolism: an overview on European data. J Inherit Metab Dis.

[16] Kido J, Matsumoto S, Häberle J (2021). Role of liver transplantation in urea cycle disorders: report from a nationwide study in Japan. J Inherit Metab Dis.

[17] Posset R, Garbade SF, Gleich F (2020). Long-term effects of medical management on growth and weight in individuals with urea cycle disorders. Sci Rep.

[18] Nakamura K, Kido J, Matsumoto S, Mitsubuchi H, Endo F (2016). Clinical manifestations and growth of patients with urea cycle disorders in Japan. J Hum Genet.

